# HAP1, a new revolutionary cell model for gene editing using CRISPR-Cas9

**DOI:** 10.3389/fcell.2023.1111488

**Published:** 2023-03-03

**Authors:** Gemma Llargués-Sistac, Laia Bonjoch, Sergi Castellvi-Bel

**Affiliations:** Institut d'Investigacions Biomèdiques August Pi i Sunyer (IDIBAPS), Gastroenterology Department, Centro de Investigación Biomédica en Red de Enfermedades Hepáticas y Digestivas (CIBEREHD), Hospital Clínic, Barcelona, Spain

**Keywords:** HAP1, CRISPR-cas9, genome-engineering, functional validation, haploidy

## Abstract

The use of next-generation sequencing (NGS) technologies has been instrumental in the characterization of the mutational landscape of complex human diseases like cancer. But despite the enormous rise in the identification of disease candidate genetic variants, their functionality is yet to be fully elucidated in order to have a clear implication in patient care. Haploid human cell models have become the tool of choice for functional gene studies, since they only contain one copy of the genome and can therefore show the unmasked phenotype of genetic variants. Over the past few years, the human near-haploid cell line HAP1 has widely been consolidated as one of the favorite cell line models for functional genetic studies. Its rapid turnover coupled with the fact that only one allele needs to be modified in order to express the subsequent desired phenotype has made this human cell line a valuable tool for gene editing by CRISPR-Cas9 technologies. This review examines the recent uses of the HAP1 cell line model in functional genetic studies and high-throughput genetic screens using the CRISPR-Cas9 system. It covers its use in an attempt to develop new and relevant disease models to further elucidate gene function, and create new ways to understand the genetic basis of human diseases. We will cover the advantages and potential of the use of CRISPR-Cas9 technology on HAP1 to easily and efficiently study the functional interpretation of gene function and human single-nucleotide genetic variants of unknown significance identified through NGS technologies, and its implications for changes in clinical practice and patient care.

## Introduction

A series of major and rapid scientific technical innovations over the past 20 years have enabled the advancement and refinement of DNA sequencing. This has opened a new window for better understanding the human genome and the genetic basis of human diseases. The rising need for high-throughput sequencing of whole genomes in any large quantity at lower costs, coupled with the development of potent and capable bioinformatic tools for data analysis, have propelled the implementation of NGS as a driving tool for translational medicine (Metzker, 2010; Beigh, 2016).

Joint efforts between scientists across the globe have made the complete sequencing of the Human Genome possible (Nurk et al., 2022). This has allowed the mapping of any sequence of interest and the identification of any existing polymorphic variants in a given genomic area. The routine implementation of high-throughput sequencing of exomes and whole genomes has provided a wealth of information on how our genes work to create a wondrous spectrum of phenotypes, including their variance across populations. NGS has enabled a deeper, but still minimal, understanding of gene function and their relationship with disease. It has allowed the discovery of many disease-causing genes and genes involved in disease predisposition (Lyon et al., 2012). This straightforward process of DNA sequencing is extremely successful in characterizing the causes of many clinical Mendelian disorders, where the mutation effect is dominant and the altered phenotype can be easily linked to the genomic change. On the other hand, complex diseases, caused by mutations in multiple genes and that have a less dominant effect, add a layer of difficulty to actually determine the implication a particular change has on a disease.

## Next-generation sequencing and genome-engineering

The revolution in massive parallel sequencing has facilitated the discovery of hundreds of thousands of new single nucleotide polymorphisms (SNPs) associated with complex human diseases, like cancer. On these grounds, despite the undeniable power of NGS, there is an unquestionable bottleneck for analysis and interpretation of data. Identified genetic variants are mostly selected according to their rarity in the general population and *in silico* prediction tools. With this deluge of sequence data provided by NGS platforms there is a growing need to assess the functional impact of each variant to unequivocally associate mutations with disease predisposition, development, or progression. The unavailability to precisely edit genes had been a major roadblock to generating novel biological insights to develop new disease prevention therapeutic strategies (Wei et al., 2014; Bonjoch et al., 2019). In this sense, the advent of new easy-to handle human cellular models that enable a robust genomic editing in a high-throughput manner, is key to unravelling the functional consequences of SNP and their role in human diseases in order to have a clinical impact.

Nuclease-assisted gene-editing techniques have overcome this barrier and allow editing of any desired genomic site. The core techniques currently utilized for gene editing are clustered regularly interspaced short palindromic repeats (CRISPR)—CRISPR-associated protein 9 (Cas9)(CRISPR-Cas9), transcription activator-like effector nucleases (TALENs), zinc-finger nucleases (ZFN), and meganucleases (MegNs) (Gaj et al., 2016; Porteus, 2016; Khalil, 2020).

## Genome engineering using the CRISPR-Cas system

The ground-breaking method in the genomic engineering field has been the CRISPR-Cas system. This adaptive ancient bacterial immune system recognizes DNA through RNA-DNA hybridization. The complex consists of a guide RNA (gRNA), complementary to the target DNA, and a Cas protein nuclease. There are different classes of Cas proteins (Makarova et al., 2018), but the current most commonly used is the type II nuclease Cas9, derived from *Streptococcus pyogenes*. Upon DNA recognition and binding, Cas9 nuclease domains are activated, generating DNA double-strand breaks (DBSs) and triggering the endogenous cellular DNA repair mechanisms (Jinek et al., 2012; Cong et al., 2013; Esvelt et al., 2013; Ran et al., 2013). Genome editing is achieved through the repair of the resulting DSBs. The cleaved DNA can be repaired through the error-prone, non-homologous end-joining (NHEJ) mechanism, or the homology-directed repair (HDR) pathway, which enables the introduction of specific point mutations or insertion of a desired sequence. Therefore, depending on the DNA repair pathway triggered, the CRISPR-Cas9 system can be used to generate gene knock-outs (KO) or gene knock-ins (KI).

### Optimization and development of more precise strategies

Since its discovery and development, the CRISPR-Cas9 system has been improved to increase gene editing precision and efficiency. It has become the genome-editing technique of choice amongst other nuclease-based functional genomic approaches due to its unprecedented ease to modify the genome of a wide variety of organisms, from animals to plants.

Its incessant application in the field has resulted in its constant evolvement and optimization. A major hurdle still very much prevalent for all engineered nucleases is the risk of off-target effects, the acquisition of unintended changes at sites other than your target. This is a direct consequence of the ability of gRNA to bind DNA sequences with less than perfect complementarity (Hsu et al., 2013). One approach currently used to increase target specificity is the refinement in guide RNA engineering, RNA complex and guide selection. Through improving the gRNA spatial conformation and its ability to hybridize with target DNA, and the use of ameliorated engineered Cas9s it has been possible to substantially increase on-target activity and specificity (Mir et al., 2018). Tool websites for guide-design are available to automate the design of the best gRNA for your target DNA sequence, evaluating the guides according to their ON and OFF-target scores. Some examples of free online tools for guide and HDR template design can be found summarized in the Review from Cui et al. (2018).

### CRISPR-Cas9 for gene knock-ins

In addition to generating knock-outs or indels, a more precise form of editing is a gene knock-in (insertion). As previously mentioned, KI rely on HDR where the cells repair DSB using a DNA template with the desired genetic change of interest. The DNA template is required to be homologous to the region immediately located around the DSB, and to be delivered simultaneously as either DNA or with the gRNA complexed with the Cas9, called ribonucleoprotein complexes (RNP). Therefore, coupled with the high specificity of CRISPR-Cas, DSB can be directed to a specific genomic site and the DNA donor sequence delivered used as template to precisely make various types of genome modifications, from single nucleotide substitutions to insertions of longer DNA sequences *via* HDR (Tyumentseva et al., 2021).

The ability to precisely and effectively introduce genetic changes into a host genome is invaluable to understand gene function and to model disease-causing mutations. Despite HDR-mediated repair being more accurate than NHEJ, its efficiency is usually very low compared to NHEJ-mediated DNA repair used to generate KO, hampering its development for clinical application (Petazzi et al., 2022).

To exploit the potential of CRISPR-Cas9 KI for clinical research, several approaches have been explored to improve HDR efficiency. From the use of chemical reagents that inhibit NHEJ or enhance HDR, such as KU-0060648 and RS-1 respectively (Chu et al., 2015; Maruyama et al., 2015; Robert et al., 2015; Yu et al., 2015; Song et al., 2016), to the use of drugs that transiently arrest cell cycle (Lin et al., 2014), since HDR only occurs in the late S and G2 phases of the cell cycle. The small molecule compound RS-1 enhanced the homologous recombination activity of hRAD51, and therefore enhanced HDR. On the other hand, KU-0060648 enriches for HDR-mediated repair events by inhibiting the DNA-PKcs pathway.

### Base editors and prime editing

The discovery of new Cas proteins (Pickar-Oliver and Gersbach, 2019) and specially Cas9 protein engineering over the last few years, has diversified and improved CRISPR systems at high speed. Base editors were developed as DSB-free methods to introduce specific nucleotide changes, reducing unintended mutations and indels. By using a catalytically dead Cas9 (dCas9) or a Cas9 nickase (nCas9) coupled to deaminase enzymes, it is possible to make the direct conversion of one base to another relying on the base-excision repair (BER) system. Nowadays, cytidine base editors (CBE) (Komor et al., 2016), adenine base editors (ABE) (Gaudelli et al., 2017) and glycosylase base editors (GBE) (Zhao et al., 2021) allow C-to-T, A-to-G and C-to-G base changes, respectively. Although base editors have demonstrated a high on-target activity, they still have some limitations regarding base transversions and the editing window. In addition, such methods are unsuitable for introducing indel mutations.

One of the last cutting-edge CRISPR advances has been the Prime Editing (PE) methodology (Anzalone et al., 2019). PE utilizes an nCas9 fused with a reverse-transcriptase (RT) and an extended gRNA that encodes for both the primer binding site and the RT template sequence with the desired edits (pegRNA). The PE approach is more versatile than base editing since it allows all potential 12 nucleotide substitutions as well as modeling small indels, increasing the editing possibilities. PE has been a significant breakthrough in the gene editing field, but as with other CRISPR methods, there is still room for optimization and improvement at both pegRNA and enzymatic levels (Scholefield and Harrison, 2021).

### Delivery systems of Cas9

Tremendous efforts have been devoted to improve the efficiency of the delivery of the CRISPR-Cas9 system. There are currently three main strategies to deliver the CRISPR-Cas9 system to edit genomes. The simplest and straight-forward is the delivery of plasmids containing vectors that encode for the Cas9 protein and the gRNA. Another approach is to deliver a mixture consisting of the gRNA and the mRNA of the Cas9. The third strategy is to directly deliver the Cas9-gRNA complex (Ran et al., 2013; Liu et al., 2017). Despite the plasmid-based CRISPR-Cas9 system being the most stable of the delivery systems, it still produces off-target effects, and its introduction to the nucleus of the cells remains challenging. On the other hand, direct delivery of the Cas9 mRNA and gRNA into the cytoplasm of the target cells reduces cytotoxicity, and most importantly, allows the transient expression of the Cas9 protein, which limits the duration of the genome-editing window, subsidizing off-target effects. The main obstacle of this strategy is the relatively poor stability of mRNA. Consequently, the most widely used strategy in recent years has been the direct delivery of RNP. Amongst the many advantages this strategy confers are its rapid action, high gene efficiency, and reduced toxicity and off-target effects (Liu et al., 2017). Upon delivery to the cell’s nucleus, the RNP complex is instantly functional without the need of transcription or translation. With RNP delivery, risks of spontaneous genome integration disappear and the short active window of the complex before degradation greatly reduces off-target effects (Schumann et al., 2015).

Various physical and non-viral delivery approaches have been extensively exploited to deliver the CRISPR-Cas9 system to target cells. Amongst the most widely used are hydrodynamic injections, electroporation, nucleofection, and nanoparticles (Gori et al., 2015; Qin et al., 2015; Zuris et al., 2015; Tyumentseva et al., 2021). The efficiency of viral vectors to deliver the plasmid-based CRISPR system is compromised by its safety, a concern that is greatly reduced when using non-viral vectors. Overall, over the past decade, tremendous efforts have been made to increase the efficiency of the system and reduce off-target effects to successfully use the technique for biological research.

## Genome editing in functional studies

The development of precise and efficient genome-editing technologies has paved the way for the deciphering of the biological relevance of many genetic variants identified so far. The ability to modify genes to the precise nucleotide has made it possible for researchers to manipulate human genomes in an attempt to recapitulate particular diseases to understand their biological basis in health and disease. It is not only possible to do that on the human genome, but several model organisms have been used to functionally assess genes.

Yeast research has been pivotal in the establishment of the conceptual framework for systematic genetics, which has provided a view on the genetic landscape and molecular organization of a cell (Costanzo et al., 2010). The use of yeast, a haploid organism, enabled many advances in the field of genetics, allowing the discovery and classification of essential and non-essential genes through the performance of systematic genetic knock-out studies. Although gene essentiality is tissue and cell dependent, the characterization of such gene functionality was of extreme importance for future advancement in genetic studies (Giaever et al., 2002; Hillenmeyer et al., 2008; Ramani et al., 2012). This distinction in gene essentiality according to cell type is extremely relevant in humans. The ability to discern genes that are essential for human diseases, being genes that either drive the initiation, or progression of the disease is of utmost importance to develop clinically-relevant management strategies based on these specific genomic changes (Hart et al., 2015).

Despite technological advancements the main challenges that remain are the main research models utilized to recapitulate the genetic background of human diseases. In recent years, several attempts have been made to create relevant haploid human cell models to propel the genetic engineering field for biological research to the next level by creating relevant human models where gene function could be studied and exploited in a human-disease context.

## Haploid human cell line HAP1

HAP1 is a near-haploid human cell line derived from KBM7, a human myeloid leukemia cell line developed from a 39-year-old male patient in the blastic phase of chronic myeloid leukemia (Andersson et al., 1987). KBM7 was a mixed ploidy line, constituted by two predominant but distinct near-haploid cell populations with disomies for chromosomes 8 and 8 and 15, and a hyper-diploid one. Its cytogenetic analysis revealed that all subclones had lost chromosome Y and carried the Philadelphia translocation, t (9q;22q), characteristic of myeloid leukemias (Andersson et al., 1995; Kotecki et al., 1999). Higher ploidy clones presented a lower doubling time than their near-haploid counterparts. Upon further cultivation, cells with greater DNA content quickly overgrew the near-haploid cells and became dominant in culture (Andersson et al., 1995). From the mixed ploidy line, a haploid stable subclone, except for a disomy of chromosome 8, was isolated and further stably cultured over several months (Kotecki et al., 1999), becoming the first human (near) haploid cell line model, known nowadays as KBM7. Since then, the near haploidy of KBM7 has been widely exploited to perform gene function studies, since baring a single copy of the genome unravels the effect of any given mutation (Li and Shuai, 2017).

In an attempt by Carette et al. to induce KBM7 cell’s pluripotency by expression of *OCT4*, *SOX-2*, c-*MYC* and *KLF411*, subclones with only one subset of chromosomes were obtained (Takahashi and Yamanaka, 2006; Carette et al., 2011). This set of subclones, termed HAP1, retained the Philadelphia chromosome, no longer expressed hematopoietic markers and, unlike KBM7, grew adherently. Spectral karyotyping analysis of HAP1 by Essletzbichler et al. revealed a fusion of a 30-megabse fragment of Chromosome 15 to Chromosome 19. They efficiently employed CRISPR-Cas9 genome engineering technology to excise the disomic region from Chromosome 15, acquiring the first fully haploid human cell line, eHAP1 (Essletzbichler et al., 2014). A few advantages HAP1 confers apart from a haploid karyotype are a rapid doubling time, sustained growth at single dilutions, and ease of transfection.

In biomedical research studies, the potential of an *in vitro* cell line used as a model system depends on its ability to maintain genome stability. Genomic disparities observed between “control” and ‘treated’ cells need to be a direct consequence of the effect of your target treatment and not off-target cytogenetic mutations. Something to take into consideration when working with HAP1 is their ploidy instability, a long-recognized trait of all haploid mammalian cells (Horii and Hatada, 2015). As happened with KBM7, HAP1 was karyotypically stable in culture for up to approximately 20 passages, further culturing reduced the percentage of haploid cells in culture, which was enriched in diploid cells. This phenomenon has long been considered as spontaneous diploidization (Egli et al., 2011; Leeb et al., 2014; Yilmaz et al., 2016; Sagi & Benvenisty, 2017; Banerjee et al., 2022), but it has recently been shown that the growth properties and reduced fitness of haploid cells is what is a disadvantage amongst diploid cells present in culture. Data on HAP1 from Olbrich et al. showed that the conversion from haploid to diploid is an infrequent event, but upon occurrence, the better growth properties of diploid cells allow them to overtake the culture. The reduced fitness is in part explained by the triggering of the p53-dependent cytotoxic response which limits the fitness of mammalian cells (Olbrich et al., 2017).

To account for such differences it is crucial to ensure HAP1 cell ploidy status. Some fundamental differences between haploid and diploid HAP1 have been described (Olbrich et al., 2017, 2019; Beigl et al., 2020), hence the underlying importance of controlling the karyotype of the cells before experimentation and phenotypic characterization. If used for genetic engineering, haploid HAP1 single-cell subclones have been found to be more ploidy stable (Olbrich et al., 2017). In order to minimize interfering variables in gene function studies, it is recommended to perform haploid HAP1 cell sorting by flow cytometry (Beigl et al., 2020). An alternative haploidy selection method would be the administration of the chemical compound 10-Deacetylbaccatin-III (DAB) and Diclazuril, which have been proven capable of selecting haploidy in HAP1 cells (Olbrich et al., 2019). Olbrich et al. performed a phenotypic chemical screen in HAP1 cell line to identify compounds capable of selecting cells with lower ploidy in mixed cultures. The authors identified DAB and Diclazuril, and further studied their mechanistic effect. The compounds were found to affect the microtubule dynamics of interphase cells, extending the duration of mitosis. While haploid cells easily overcame the mitotic arrest, higher ploidy cells presented very prolonged arrests followed by cell death. Therefore, the compounds were shown to facilitate the maintenance of haploid cells by delaying the formation of the metaphase palate. (Olbrich et al., 2019).

Recently, Banerjee et al. (2022) performed a detailed analysis of cytogenetic characterization of HAP1 cell lines using the single cell-based assay M-FISH karyotyping. The group karyotypically characterized 19 CRISPR-Cas9 engineered HAP1 cells to assess their rates of chromosomal instability and explore their possible underlying causes. Sixteen out of 19 cell lines with passage numbers ranging from 10 to 35 revealed a diploidy percentage range of 2%–35%, results in accordance with a previous study from Beigl et al. (2020) which show that HAP1 cell cultures are haploidy-stable for at least 20 passages. These recent studies highlight the importance of using cell lines at low passage for biomedical research and the implementation of routine karyotype controls to avoid accumulation of mutations that can induce further genome instability.

The inherent ability of HAP1 to diploidize is one of the tremendous advantages of using HAP1. It has so far been proven to not affect engineered cells and therefore entailing the conversion of haploid into diploid human cells representing homozygotes for the genetic change of interest (Drazic et al., 2018; Oishi et al., 2018).

## Functional studies using HAP1

HAP1 is an easy and amenable model to use for research. Since its establishment, the haploid HAP1 human cell line, along with its mother cell line, has been used for a wide array of studies across fields, from cell biology to virology, metabolism, apoptosis, drug testing, genome integrity, and functional genomics to name a few (Agrotis et al., 2019; Depetter et al., 2019; Guiducci et al., 2019; Moskovskich et al., 2019; Nixon et al., 2019). A summary of studies using HAP1 is found in Table 1. The primary advantage of haploid human cell lines is the fact that genes are hemizygous and therefore, upon mutations, phenotypes are immediately exposed which enables to study the effect of specific mutations in a human-related context (Wutz, 2014). Its second most precious attribute is its doubling time, at around 48 h, and ability to efficiently grow as single-cells, which enables the rapid cultivation of the cells for experimentation.

**TABLE 1:** Summary table of functional studies using HAP1. Summary of some of the genes studied using the haploid human cell line model HAP1, classified by field, gene studied and method of CRISPR-Cas9. The full table can be found in Supplementary Table S1.

Field	Gene studied	CRISPR-CAS9	References
Oncology	BRCA1	HDR library SGE	Findlay et al. (2014) (DOI: 10.1038/nature13695)
		Saturation genome editing	Findlay et al. (2018) (DOI: 10.1038/s41586-018-0461-z)
	genome-wide lethality screen	plasmid vector delivery	Gisler et al. (2020) (DOI: 10.1371/journal.pone.0227592)
	FANCC	GeCKO library	Moder et al. (2017) (DOI: 10.1038/s41467-017-01439-x)
	MSH2	lentiviral plasmid - saturation mutagenesis	Jia et al. (2021) (DOI: 10.1016/j.ajhg.2020.12.003)
Virology	E3 ubiquitin ligase	plasmid vector delivery	Liu and Moss (2016) (DOI: 10.1128/JVI.00869-16)
	DAG1	*custom-made KO*	Acciani et al. (2017) (DOI: 10.1128/JVI.00574-17)
	TMEM16F, XKR8	*custom-made KO*	Acciani et al. (2021) (DOI: 10.1128/JVI.01165-21)
Epigenetics	FOXO3	plasmid vector delivery	Grossi et al. (2018) (DOI: 10.1093/nar/gky331)
	FBX025	*custom-made KO*	Teixeira et al. (2017) (DOI: 10.1016/j.abb.2017.04.003)
	HDAC1, HDAC2	plasmid vector delivery	Sawai et al. (2017) (DOI: 10.1074/jbc.M117.803171)
Immunology	Suppressor of IKKepsilon	*custom-made KO*	Sonnenschein et al. (2018) (10.1002/2211-5463.12454)
	CD46, CD55, CD59	plasmid vector delivery	Thielen et al. (2018) (DOI: 10.1016/j.jim.2018.02.004)
	MR1	plasmid vector delivery	Kulicke et al. (2022) (DOI: 10.1016/j.jbc.2021.101542)
Cell/Molecular Biology	ATF3, ATF4, NOXA	plasmid vector delivery	Núñez-Vázquez et al. (2020) (DOI: 10.1111/febs.15480)
	genome-wide	GeCKO lentiviral pooled libraries	Chidawanyika et al. (2018) (DOI: 10.1038/s41420-018-0135-5)
	TRAF2	*custom-made KO*	Palumbo et al. (2022) (DOI: 10.1016/j.biocel.2022.106193)
Metabolism	TMEM189	*custom-made KO*	Werner et al. (2020) (DOI: 10.1073/pnas.1917461117)
	FASN, SPRING	plasmid vector delivery	Loregger et al. (2020) (DOI: 10.1038/s41467-020-14811-1)
	PEDS1	*custom-made KO*	Werner et al. (2022) (DOI: 10.1007/s00018-022-04238-w)
Drug Validation	GSK-3β	*custom-made KO*	Kauffman et al. (2020) (DOI: 10.1016/j.leukres.2020.106464)
	HDAC6	*custom-made KO*	Depetter et al. (2019) (DOI: 10.1002/ijc.32169)
	ENT1, ENT2	*custom-made K.O.*	Jia et al. (2021) (DOI: 10.1007/s13318-021-00703-2)
Proteomics	UPS and 41 other genes	lentiGuide-Puro plasmid for sgRNA	Hundley and Toczyski (2021) (DOI: 10.1016/j.xpro.2021.100685)
Functional Genomics	136 genes	*custom-made KO*	Smits et al. (2019) (DOI: 10.1038/s41592-019-0614-5)

The first use of human haploid cell lines consisted of forward genetic screens using KBM7. For the first time a human haploid cell line was easing the performance of biological research to study the functionality of human genes. The first haploid genetic screen in human cells used KBM7 to develop a screening method to generate null alleles. Insertional mutagenesis was chosen as the approach of interest to inactivate genes due to its high level of mutagenicity across species, and because the DNA sequences integrated in the genome can be used as tags to identify the disrupted gene (Carette et al., 2009). As previously mentioned, subsequent studies using haploid genetic screens by Carette et al. (2011) led to the generation of HAP1 as a failed attempt to induce pluripotency in KBM7. The first genome wide study using HAP1 was conducted using a retroviral gene-trap vector to mutagenize early-passage HAP1 cells, which enabled the identification of essential host factors for the entry pathway of Ebola virus. With the ease haploidy confers for forward genetic experiments, as only one allele needs to be mutated, another approach performed using HAP1 enabled the identification of essential factors for Lassa viral infection (Jae et al., 2014).

Due to their said relative rapid growth and ease of transfection, HAP1 cells have been used extensively in CRISPR-Cas9 screens. Due to their great amenability, several researchers have exploited HAP1s’ culture properties to conduct high-throughput functional screens to enhance their study’s potential. To facilitate the wide application of HAP1, some researchers have developed protocols to use HAP1 as a cell model to perform various gene-editing studies. Lackner et al. (2015) developed a protocol for gene tagging based on CRISPR-Cas9 using HAP1, allowing the authors to establish a strategy to endogenously tag genes at both the N and C terminus, without the integration of adjacent plasmid sequence. More recently, Hundley and others developed a detailed protocol to perform high-throughput system-specific chemical-genetic CRISPR-Cas9 screens in the human HAP1 cell line using a pathway-specific library or sub-genomic library (Hundley and Toczyski, 2021).

### Studies using CRISPR-Cas9 system to edit HAP1

The exuberant development of genome-editing techniques has revolutionized research on the human genome, allowing the generation and introduction of genetic variants in a wide variety of cell models for their functional characterization at a great speed. General approaches to generating informative functional data are still too slow and expensive to meet the growing need. The possibility to apply the CRISPR-Cas9 technique to precisely edit the genome at the nucleotide level offers a new avenue in research to start functionally characterizing mutations relevant to complex human diseases like cancer. Multiplex assays of variant effect allow the engineering and testing of variants in a pooled format, making them highly scalable, drastically reducing cost and minimizing sample processing (Gasperini et al., 2016; Weile and Roth, 2018; Findlay, 2021).

In the past decade, HAP1 has been widely established as a cell line model for a broad range of gene-editing studies, from forward genetic screens to high-throughput CRISPR-Cas9 screens. Despite being a leukemia cell line, it has become the cell model of choice across different scientific fields due to its ease of transfection and acquisition of single-cell clones, which allows results in a time-efficient manner. In addition, the utilization of the CRISPR-Cas9 system on a haploid human cell model like HAP1 can facilitate the gene editing process and increase editing efficiencies, since only one allele needs to be modified. A few examples of studies using HAP1 are summarized below.

#### Oncology

Back in 2014, Findlay and others demonstrated the feasibility of generating and functionally analyzing thousands of variants of programmed genome edits in a single experiment using saturation mutagenesis in HAP1. The authors coupled CRISPR/Cas9 RNA-guided cleavage with multiplex HDR using a complex library of donor templates to assess all possible SNVs so far identified on exon 18 of *BRCA1* in breast cancer (Findlay et al., 2014). A of couple years later the group presented saturation genome editing (SGE) as an innovative approach to assess the pathogenicity of *BRCA1* variants. The researchers used HAP1 to assess the clinical significance of nearly 4000 SNV in 13 exons encoding functionally crucial domains in *BRCA1* (Findlay et al., 2018). Similarly, Radford et al. used HAP1 to perform SGE of *DDX3X* gene to study the functional impact of 12776 SNV (Radford et al., 2022). In an attempt to prospectively and systematically measure the functional impact of missense variants in *MSH2*, one of the major Lynch syndrome genes, Jia et al. (2021) performed a massively parallel screen in human cells (including HAP1) to identify loss-of-function (LOF) missense variants in the DNA mismatch repair factor *MSH2*.

The CRISPR-based alternative technologies base and prime editing have emerged as tools to continue to improve the creation of programmed variants. Recently, the first large-scale base editor screens were published using HAP1, where Hanna et al. (2021) assessed more than 52,000 variants enabling the discovery of LOF variants in *BRCA1* and *BRCA2* genes and mapping protein residues where variants confer sensitivity or resistance to targeted therapies. In a similar high-throughput manner, BE was used in HAP1 to create missense variants across 86 DNA damage response genes, discovering functionally critical loss- and gain-of-function protein domains and providing evidence for reclassification of variants of uncertain significance for breast cancer (Cuella-Martin et al., 2021).

For a more therapeutic target based approach, Billaud *et al.* developed a rapid functional assay using HAP1 to characterize the functional implication of variants in tumor suppressor genes *BRCA1*, *BRCA2* and *POLE* to patients’ response to therapy (Billaud et al., 2021). Alternatively, Neggers *et al.* created a CRISPR-mediated NHEJ repair genetic screening approach to rapidly derive and identify drug-resistance mutations in essential genes (gain of function mutations) (Neggers et al., 2018). To study the role of kinase deregulation in tumor initiation and/or progression, Robinson-Garcia *et al.* developed a CRISPR-Cas9 systematic approach to identify synthetic lethal interactions for kinase deficiencies to different DNA-damaging chemotherapeutic agents using HAP1 (Robinson-Garcia et al., 2019). Another technique to exploit synthetic lethality in HAP1 was conducted by Gisler et al., by performing a genome-wide screen for gene disruptors that could result in resistance to pharmacological inhibition of BMI1 on HAP1 by exposing mutagenized HAP1 to low concentrations of the BMI1-inhibitor PTC-318 (Gisler et al., 2020).

Other diseases in the field of oncology have also benefited from the scalability of experiments when performed with HAP1. Moder et al. introduced an approach to systematically study synthetic viability using HAP1, where a genome-wide screen was performed on cells with LOF for the Fanconi anemia complementation group C (FANCC), and used insertional mutagenesis coupled with genome-wide CRISPR libraries to identify genetic suppressor interactions for Fanconi anemia (Moder et al., 2017). In a similar way, a genome wide genetic screen on HAP1 was conducted to uncover tumor-intrinsic genes that regulate tumor killing by major histocompatibility complex-unrestricted cytotoxic lymphocytes (Menasche et al., 2020).

#### Virology

In the virology field Guedán et al. also exploited HAP1 haploidy to generate knock-outs for *PKD1* and *PKD2* genes using CRISPR-Cas9 to study Human Rhinovirus replication. Although the authors did not identify the molecular mechanisms by which PKD regulates viral replication, the use of HAP1 *PKD* knock-out cell lines allowed the study of small-molecule inhibitors targeting *PKD*, suggesting it as a novel antiviral target for drug discovery (Guedán et al., 2017).

#### Immunology

In the immunology field, Sonnenschein et al. used HAP1 to create CRISPR-Cas9 knockout cells to study the type I interferon response of the innate immune system. The authors wanted to study the interaction between the suppressor of IKKepsilon (SIKE) and TANK-binding kinase 1, associated with the activation of interferon regulatory factors to initiate a type I interferon response. Using the CRISPR-cas9 system, expression of *SIKE* was knocked out in HAP1 to investigate the protein interactions of SIKE during the innate immune response, which enabled the identification of the direct interaction of SIKE with the cytoskeleton of cells to promote cytoskeletal rearrangements to initiate cell movement either for phagocytosis or migration (Sonnenschein et al., 2018).

The complement system is a part of the immune system that plays a pivotal role in the innate defence against pathogens and host homeostasis. The complement is initiated upon sensing danger signals that are caused by confirmational changes of molecular complexes, initiating a tightly regulated cascade of enzymatic reactions to modulate the adaptative immune response and regulate host homeostasis. Thielen et al. generated knockout HAP1 cells for the complement regulatory proteins CD46, CD55 and CD59 to investigate the complement activation and regulation of human cells (Thielen et al., 2018).

#### Epigenetics

HAP1 has also been used in the epigenetic field to study SUMOylation. Important cellular processes like transcription, nuclear transport and maintenance of genome integrity are regulated by the reversible post-translational modification of SUMOylation. Rodríguez-Castañeda et al. used HAP1 to generate CRISPR-Cas9 knock-outs for *SENP1* and *CHD3* genes by generating frameshift mutations in the coding region of each gene. They reported a novel level of coordinated gene expression control between *SENP1* and *CHD3* (Rodríguez-Castañeda et al., 2018).

#### Cell biology

In a more high-throughput manner Chidawanyika et al. conducted a genome-wide CRISPR-Cas9 loss of function screen against HAP1 cells to study the stress-induced endoplasmatic reticulum (ER) cell death. With the aim to identify genes essential for cell death induced by the classical pharmacological ER stressors thapsigargin, tunicamycin, and brefeldin, the authors used pooled CRISPR-Cas9 human libraries to perform thorough and unbiased loss-of function screen against thapsigargin, tunicamycin, and brefeldin. With this approach the authors were able to identify a novel essential gene, *SEC24A*, for thapsigargin-induced cytotoxicity (Chidawanyika et al., 2018).

Researchers studying the translational apparatus developed a novel method for targeting one of the two dedicated RNA components of the re-coding machinery, the selenocysteine-tRNA. In their study, Vindry et al. developed a novel CRISPR strategy based on Cas9-sgRNA ribonucleoproteins loaded with murine leukaemia virus-like particles (VLPs) to inactivate the Sec-tRNA gene in human cell lines, HAP1 amongst them. The authors successfully reduced selenoprotein expression by the development of CRISPR-Cas9-VLP, important for regulating the activation of selenoproteins (Vindry et al., 2019).

#### Metabolism

In the metabolism field, HAP1 has also been used to study calcium activity in eukaryotic cellular processes. Karakus et al. used HAP1 to functionally assess for the first time the molecular function of a newly identified negative regulator of intracellular calcium signalling, SLC10A7. The authors used *SLC10A7* KO HAP1 cells to functionally study the gene and its genomic variants to further clarify the pathogenesis of *SLC10A7*-associated diseases (Karakus et al., 2020).

The wide range of studies from various areas in biological research referenced here using HAP1 are proof for the broad applicability of HAP1 for genome engineering studies. Researchers face the challenge to recreate human disease in animal models, and such a thing is in itself extremely laborious. As technical innovations help improve the methods which researchers use to better understand human diseases, new more amendable human cell models are being generated to better recapitulate human diseases for their deeper understanding. In this case, as previously mentioned, the major benefit of HAP1 is its stable haploid karyotype, which only requires the modification of only one allele to study the effect of your mutation/change of interest. With its spontaneous diploidization over subsequent culturing, the edited allele will duplicate and give rise to the diploid edited state. The power of haploid human cells can aid in the functional validation of many variants that have been identified through NGS, and help prove their functional roles as well as implication in disease. Nevertheless, some drawbacks should also be considered. As with most cell lines of tumor origin, the HAP1 genetic background must be taken into account when studying certain cell functions or molecular pathways, such as the already described *TP53* alteration (Moder et al., 2017). Also, its ploidy instability could be a limitation for prolonged *in-vitro* cell culturing, such as assessing the mutational burden over time when focusing on those genes involved in DNA damage and repair pathways.

## Conclusion

In this review, the use of HAP1 in the recent years to perform genome-engineering using the CRISPR-Cas9 system has been explored. All the studies cited here demonstrate the power of CRISPR-Cas9 on HAP1 to study the functional consequences of genomic alterations. Again, researchers need powerful and relevant human models to study diseases. The haploidy of HAP1 offers a tremendous advantage amongst diploid human cells when generating genetic variants to elucidate the implication of specific genomic changes in the development and/or progression of diseases. With more precise genome engineering techniques and the use of relevant human cell models, researchers will be able to precisely recapitulate human diseases. Models will enable to study the function of such genomic changes with the aim to identify clinically-relevant molecular events related to disease to develop new management strategies based on these specific genomic changes.

## Supplementary material

Supplementary Table S1 Summary table of the genes studied using HAP1 since its establishment.
